# Incidence o fmicrobial colonization in coronoray care unit

**DOI:** 10.1186/1753-6561-5-S6-P75

**Published:** 2011-06-29

**Authors:** A Spyrou, J Triklis, C Panagiotou

**Affiliations:** 1 Coronary Care Unit, Onassis Cardiac Surgery Center, Athens, Greece; 2 Emergency Department, Onassis Cardiac Surgery Center, Athens, Greece

## Introduction / objectives

Nosocomial infections in patients admitted in coronary intensive care unit (CCU) are frequently caused by potentially pathogen microorganism (PPM). The aim of the present study is 1) to determine the incidence of PPM in patients admitted in our CCU the last year 2) to identify the risk factors for colonization with PPM.

## Methods

Electronic medical records of all patients without previous infection who hospitalized in CCU unit from January since December 2010 were reviewed. During hospitalization, specimens were taken from the nasopharynx, blood and urine cultures and if applicable from the central or peripherals lines.

## Results

49 patients were included in the study with mean age 63,73yrs (SD=15.45). 64% of the participants were colonized with PPM. The most common isolated pathogens were Staph.Epidermidis (36.7%), Klebsiella Pneumoniae (32.6%), Pseudomonas Aeruginosa (10.2%), Candida Albicans (8.2%) και MRSA (4.1%). Risk factors for colonization with PPM were found the duration of stay in CCU (Anova test, F= 5.008, p=.004) and the high levels of urea and creatinine (Anova test, F= 4.502, p=.039).

## Conclusion

The rates of PPM were significant high. Proper attention should be given in the risk factors that were found to be correlated.

## Disclosure of interest

None declared.

